# Identifying the core concepts of pharmacology education

**DOI:** 10.1002/prp2.836

**Published:** 2021-07-21

**Authors:** Paul J. White, Elizabeth A. Davis, Marina Santiago, Tom Angelo, Alison Shield, Anna‐Marie Babey, Barbara Kemp‐Harper, Gregg Maynard, Hesham S. Al‐Sallami, Ian F. Musgrave, Lynette B. Fernandes, Suong N. T. Ngo, Tina Hinton

**Affiliations:** ^1^ Faculty of Pharmacy and Pharmaceutical Sciences Monash University Parkville VIC Australia; ^2^ Department of Pharmacology Monash University Clayton VIC Australia; ^3^ Department of Biomedical Sciences Macquarie University Sydney NSW Australia; ^4^ Eshelman School of Pharmacy University of North Carolina Chapel Hill NC USA; ^5^ Discipline of Pharmacy Faculty of Health University of Canberra Bruce Canberra ACT Australia; ^6^ Faculty of Medicine and Health University of New England Armidale NSW Australia; ^7^ School of Biomedical Sciences Charles Sturt University Wagga NSW Australia; ^8^ School of Pharmacy University of Otago Dunedin New Zealand; ^9^ Adelaide Medical School The University of Adelaide Adelaide SA Australia; ^10^ School of Biomedical Sciences The University of Western Australia Crawley WA Australia; ^11^ Faculty of Sciences The University of Adelaide Roseworthy SA Australia; ^12^ School of Medical Sciences Faculty of Medicine and Health Sciences The University of Sydney Sydney NSW Australia

**Keywords:** concept inventory, core concept, pharmacology education, postgraduate education, undergraduate education

## Abstract

Pharmacology education currently lacks an agreed knowledge curriculum. Evidence from physics and biology education indicates that core concepts are useful and effective structures around which such a curriculum can be designed to facilitate student learning. Building on previous work, we developed a novel, criterion‐based method to identify the core concepts of pharmacology education. Five novel criteria were developed, based on a literature search, to separate core concepts in pharmacology from topics and facts. Core concepts were agreed to be *big ideas*, *enduring*, *difficult*, *applicable across contexts*, *and useful to solve problems*. An exploratory survey of 33 pharmacology educators from Australia and New Zealand produced 109 terms, which were reduced to a working list of 26 concepts during an online workshop. Next, an expert group of 12 educators refined the working list to 19 concepts, by applying the five criteria and consolidating synonyms, and added three additional concepts that emerged during discussions. A confirmatory survey of a larger group resulted in 17 core concepts of pharmacology education. This list may be useful for educators to evaluate existing curricula, design new curricula, and to inform the development of a concept inventory to test attainment of the core concepts in pharmacology.

## Graphical abstract

Over 50 pharmacology educators from Australia and New Zealand worked together to identify the core concepts of pharmacology education. The figure shows the 19 concepts identified and the process taken to produce this list

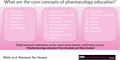

**Abbreviations**
ASCEPT: Australasian Society of Clinical and Experimental Pharmacologists and Toxicologists
CC‐PEG: core concepts in pharmacology expert group

## 1 INTRODUCTION

In the early 1990s, a startling, and very distressing discovery sparked an ongoing revolution in undergraduate physics education and subsequently biology education. Hestenes and his colleagues demonstrated that even the best prepared fourth‐year students majoring in physics in elite US institutions were unable to apply key concepts. Their seminal work developing^1^ and evaluating^2^ the Force Concept Inventory found, for example, that while 80% of students could recite Newton's third law at the beginning of a course, less than 15% fully understood it at the end. These findings suggested that students had not developed the effective conceptual frameworks essential to human learning and application of knowledge.^3^ Fortunately, the ability to measure students’ conceptual understanding, as well as to identify the gap between what has been taught versus what was learned, has resulted in substantial advances in physics and biology education that have improved learning of these critical disciplines. The strides these educators made have yet to be applied systematically to the discipline of pharmacology.

We argue that a consensus list of the core concepts of pharmacology education is well overdue. Over 30 years of educational research have established that the identification of *core concepts*, and the development of *concept inventories* to assess them, can be transformative innovations. Physics education innovators^1^, ^4^, ^5^ were joined by a large, coordinated approach in biology education in the early 2000s. The US National Science Foundation and American Association of Advancement in Science brought together 500 educators to produce five core concepts in biology within a Vision and Change Manifesto.^6^ Subsequently, resources have been developed to help biology educators to incorporate the teaching and assessment of core concepts into their curricula.^7^ Sub‐disciplines within biology, including physiology and microbiology,^7^, ^8^, ^9^, ^10^, ^11^ and other disciplines such as information technology^12^ and engineering statistics^13^ have identified core concepts. In contrast, pharmacology education currently lacks an agreed set of core concepts.

### 1.1 Why do we need core concepts in pharmacology?

Efforts to identify core concepts within curricula have been motivated by a range of factors, all of which are relevant to pharmacology educators. An analysis of core concept development studies reveals motivations ranging from a desire for an agreed curriculum to the use of core concepts and concept inventories to achieve transformative change. A common or overarching theme was expressed by physiologist Joel Michael:

“Teaching and learning should focus on core concepts and deep learning, not the accumulation of ever more facts.”^14^

Primary motivations in psychology,^15^, ^16^ information technology/cybersecurity,^17^ dietetics,^18^ biology,^8^ and mathematics^19^ centered around providing the discipline with an agreed curricular conceptual focus and to reduce curricular variability, and concept inventories were developed to assess and compare attainment of this agreed curriculum.^4^, ^12^, ^20^, ^21^, ^22^, ^23^, ^24^ The contents of courses and curricula typically include a mix of topics, facts, skills, contexts, and concepts. Core concepts may prove useful as anchors for the conceptual content.

“Mathematics education is mired in the “math wars” between “back‐to‐basics” advocates and “guided‐discovery” believers. There is no possibility of any resolution to this contest between competing faiths without scientific evidence of what works and what doesn’t.”^19^

Deeper learning aspirations included providing students with the vocabulary of the discipline^16^ and helping students develop conceptual frameworks^25^, ^26^:

“The critical goal of teaching is to help students develop a **conceptual framework** that embraces relevant facts and concepts rather than isolated bits of knowledge.”^16^

In the biological sciences in particular, core concepts provide a focus on what is important and encourage depth in the face of exponential growth in knowledge^7^, ^14^, ^19^:

“The knowledge explosion is alive and well in physiology…the focus on learning more “content” does not help students understand physiological principles.”^27^

Health professional educators have specific needs in integrating knowledge from a range of primary disciplines^7^:

“Due to the advances in technology and therapeutics the general care nurses must possess an “amalgam of knowledge” from different areas.”

As concept inventories in physics became established and demonstrated that most current graduates had not developed deep understanding of concepts or skills in applying concepts,^7^, ^28^ core concepts and related inventories in other disciplines were developed to help instructors know whether their students had attained conceptual understanding^29^ and prompt new teaching approaches.^25^ Some educators within biology have actually implemented a core concepts‐based curriculum^7^:

“The biology community is largely in agreement that undergraduate biology majors should master the core concepts.”

In physics and other disciplines, the identification, and destigmitization of *“common sense beliefs*—*alternative conceptions”* that must be identified and overcome for students to learn core concepts has been central to their development.

“Every student begins physics with a well‐established system of common‐sense beliefs about how the physical world works derived from years of personal experience. … Instruction that does not take them into account is almost totally ineffective.”^1^

Finally, some researchers are referring to increasing community expectations of accountability of university educators and the need for social good to improve the public's knowledge and standard of discourse:

“Several forces are now driving change in undergraduate astronomy education in the USA. These include: a call for postsecondary faculty to document the effectiveness of their teaching.”^5^

We argue that all of the above motivations are relevant to pharmacology. The multi‐disciplinary nature of pharmacology, with roots in biology, chemistry, and physics, and the enormous body of knowledge in this field means that educators struggle to decide what to teach and assess. The authors independently rated the above drivers for core concept development as they relate to pharmacology education. Our consensus view was as follows, from most important as #1:

1. Core concepts provide focus on what is important and encourage depth in the face of exponential growth in knowledge
2. Teaching and learning should focus on core concepts and deep learning, not on the accumulation of ever more facts.
3. Core concepts provide students with the vocabulary of the discipline and help students to develop conceptual frameworks.
4. Core concepts provide the discipline with an agreed conceptual curriculum, reducing curricular variability, and concept inventories can be developed to assess and compare attainment of this agreed curriculum.
5. Core concepts and inventories to assess their attainment help instructors to know whether their students have attained conceptual understanding and prompt new teaching approaches.
6. Health professional educators have specific needs for integrating knowledge from a range of primary disciplines, which is assisted by core concept development.
7. Core concepts and inventories to assess their attainment are required for the identification and destigmitization of *“common sense beliefs*—*alternative conceptions”* that must be overcome for students to learn core concepts.
8. Most current graduates may not have developed deep understanding and skill in applying concepts.
9. Core concepts provide transparency of student outcomes to address increasing community expectations of university educators and the need for social good.

### 1.2 Professional considerations

Pharmacology education encompasses a spectrum of contexts from science and biomedical science through to medicine, pharmacy, nursing, dentistry, and other health professions. Health professionals require a strong foundation of knowledge across multiple disciplines in order to make appropriate clinical decisions. Studies have shown that the cognitive integration of basic sciences knowledge with clinical training leads to better clinical reasoning in health professional students. However, as Rikers and colleagues point out, “*knowledge encapsulation can only be accomplished when there is something to encapsulate”*.^30^ Health professional educators therefore have specific needs for integrating knowledge from a range of primary disciplines.^31^

### 1.3 How are core concepts defined?

As motivations for the development of core concepts have evolved, so too have the definitions used to describe these constructs. An early study^16^ illustrated the challenge of separating core concepts from topics, skills, and facts: *“After reviewing the task of imposing order on what at times seemed to be psychology word salad*, *more objective criteria appeared impossible*.*”* but later work has made good progress in this area. Some common themes emerged in definitions used in the development of core concepts. An early contention was that core concepts were important for all graduates to know.^7^, ^15^, ^16^, ^20^, ^25^ *“Big ideas”*, proposed by Wiggins et al.,^32^ was used as a framing concept in a number of core concepts studies in biology.^26^, ^33^, ^34^ The enduring or timeless^17^, ^18^, ^35^ nature of core concepts was a common theme, sometimes expressed as *“what we want every student to understand (be able to use) long after the course is completed”*.^14^ Characteristics of core concepts ranged from difficult for students to understand^17^ to transferability: *“having great transfer value horizontally (across subjects) and vertically (through the years in later courses)”*.^26^ Perhaps most important, but not always explicit was the property of core concepts that makes them *“useful to solve problems or predict outcomes”*,^8^, ^14^ a motivation clearly integral to the use of concept inventory development.^1^, ^23^, ^25^

### 1.4 Core concepts and threshold concepts

The authors note the important body of work originating from the seminal proposition from Myer and Land that some concepts are so vital that their attainment can be responsible for *“transforming the internal view of subject matter or part thereof”*.^36^ We view threshold concepts as a subset of core concepts, and anticipate a later study identifying threshold concepts in pharmacology.

### 1.5 Methods for core concept development

A range of approaches have been developed to identify core concepts. These commonly have some or all elements of a Delphi Exercise, involving a large group of participants completing cycles of surveying and refinement based on early responses.^7^, ^10^, ^15^, ^17^, ^25^, ^26^ Expert groups or sub‐groups have been utilized to analyse survey responses and extract core concepts.^7^, ^8^ Core concepts have also been extracted from textbooks, either via page‐by‐page expert analysis^16^, ^25^ or more recently via data mining techniques.^37^

The aim of this study was to develop proof‐of‐concept for a criterion‐based method to identify, define, and unpack the core concepts of pharmacology as defined by pharmacology educators in Australia and New Zealand. This will have the dual benefit of providing the concepts for which concept inventories can be produced and providing the first steps toward a framework for the evidence‐based curricular design of pharmacology courses. We note that an integrated discipline such as pharmacology requires a rigorous approach to the identification of core concepts for concept inventory development, and therefore we regard this as the first step to a concept inventory for pharmacology education.

There are emerging and useful indicative curricula for educators to follow, including extensive work from the British Pharmacological Society, and International Union of Pharmacology Education Project, however, these do not specify core concepts. Although there is an online list of core concepts in pharmacology through the Aquifer Sciences^38^ initiative in the USA, there is no published and validated set of core concepts in the field of pharmacology education.

## 2 MATERIALS AND METHODS

### 2.1 Overall study design

**FIGURE 1:**
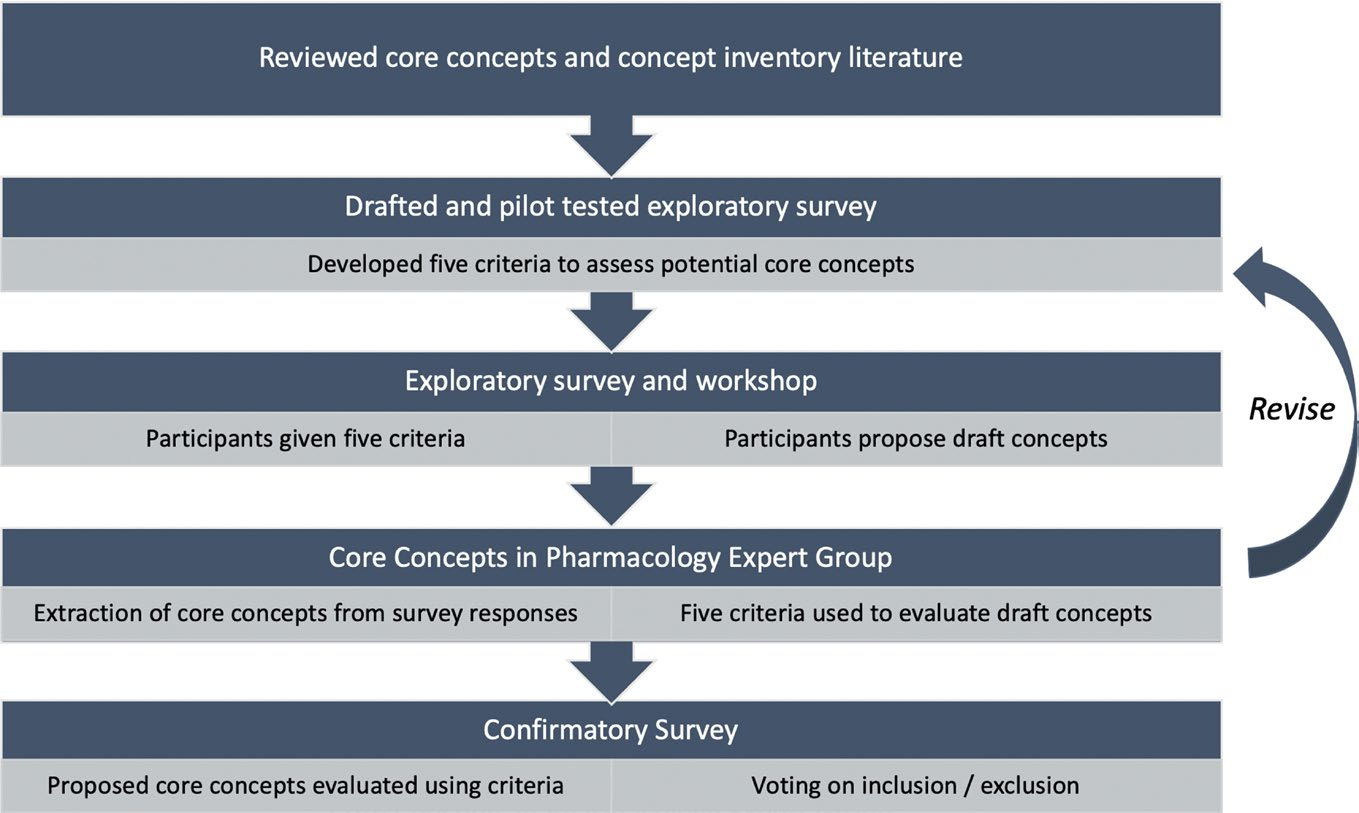
Study design and description of the five stages used

The project was conducted in five phases as shown in Figure 1.

### 2.2 Ethics approval

Project ID 22727 “Core concepts” was approved as low risk by the Monash University Human Research Ethics Committee.

### 2.3 Pilot survey: how to elicit concepts from pharmacology educators

An exploratory survey with nine respondents at the British Pharmacological Society meeting in Edinburgh 2019 was used to develop and refine the wording of the survey. TA and PW constructed a brief survey that defined a core concept as “*one which experts in a domain agree is fundamental*, *enduring and useful*, *and*, *therefore*, *necessary to understand*, *remember and apply”*. The key question asked of participants: “*3–5 years after your students graduate*, *which few concepts from your pharmacology subject*/*course would you expect them still to remember*, *understand deeply*, *and apply?”*. The responses revealed a need to further explain and define core concepts, as approximately half the responses were beyond the scope of pharmacology and/or were loosely phrased (“*bench to bedside*”), or too specific or narrow (“*care in administering corticosteroids*”), or were topics (“*regulatory affairs*”) rather than concepts. Simply asking pharmacology educators *“to nominate core concepts”* without providing criteria with which to guide them was ineffective. Like Zechmeister and colleagues 20 years earlier, we realized the difficulty of “*imposing order on what at times seemed to be a…word salad”*,^16^ and therefore set out to develop criteria that could be used to rigorously discriminate between core concepts and other survey responses.

### 2.4 Core concepts criteria

As we sought to fill the gap in core concepts and concept inventory methodology: explicit and instructive criteria to separate core concepts from other responses, TA and PW first analysed the literature. Our objective was to identify criteria that could be consistently applied by educators in the field to discriminate between core concepts in pharmacology and ideas that were not core concepts in pharmacology. To our knowledge, this is the first study to systematically apply a set of criteria to the development of core concepts.

Of the definitions used in previous studies, we felt that *“important to know”* was too vague to be discriminating. *Big ideas*, first proposed by Wiggins and McTighe,^32^ was chosen as a framing concept. After our pilot survey at the British Pharmacological Society meeting where many of the responses seemed more like facts or topics than concepts, we added “*not topics or facts*”. However, we found that this criterion was not necessary beyond the initial survey stage. We therefore adopted the following five defining criteria: *enduring*,^17^, ^18^, ^35^ *difficult* for students to understand,^17^ *applicable in multiple contexts* ^26^ and *having utility for students to explain*/*predict outcomes*.^14^ We note that while these criteria are expressed with the student who is engaged with them in mind; the criteria are designed primarily for educators who are tasked with identifying the core concepts of a discipline. We used brief phrases to guide the respondent as to the meaning of each criterion, as follows:

### 2.5 Core concepts

∙ can be applied in multiple contexts
  ○ are broadly applicable
∙ have utility
  ○ can explain and/or predict outcomes
∙ are big, critical, powerful ideas
  ○ often a key part of a discipline's conceptual framework and structure
∙ are enduring
  ○ unlikely to change over a generation
∙ are difficult
  ○ is this concept difficult for students?

### 2.6 Initial survey and related workshop

The survey (Data S1) was developed after a review of methods to identify core concepts in other disciplines. The survey initially consisted of three parts: (i) information about core concepts; (ii) a series of demographic questions and (iii) the key question *“Imagine your current*/*recent pharmacology students 3 to 5 years after their graduation*. *What few essential core concepts would you expect them to remember*, *understand deeply*, *and apply effectively in their professional work?”*.

“Please list a few—ideally between 3 and 7—core concepts that are foundational for pharmacology students in the text box below. Feel free to write as much or as little as you wish about your core concepts.”

We refined the survey using the criteria for core concepts shown above, and included that guidance in the survey.

“Your draft concepts should be big ideas that are useful to solve problems and enduring, and they should not be topics or facts.”

As a result of this experience, and similar outcomes from a pharmacy core concepts survey in 2019, we formed a view that individual responses—produced without time to think and the opportunity to discuss with peers—would need to be refined by groups of experts working together. We decided that expert groups would be required to extract the concepts from survey responses.

The refined survey was conducted within a workshop held as part of the Australasian Society of Clinical and Experimental Pharmacologists and Toxicologists (ASCEPT) annual scientific meeting in November 2020. There were 41 participants who completed the survey, and a subset of 23 participants then engaged in a range of activities over the 3‐hour workshop to refine their ideas on the core concepts of pharmacology. Respondents taught (in order of frequency) biomedical science, science, medical, postgraduate research, pharmacy, nursing and dentistry students, and 92% had PhDs as their highest earned academic degree. Approximately one‐third (35%) of respondents had been teaching for more than 20 years and 71% were female.

Participants were assigned randomly to sub‐groups of 4–6, and each sub‐group was tasked with identifying up to ten draft concepts and then rating each concept using the criteria described above. At the end of the session, the sub‐groups compared lists and an initial draft set of core concepts was produced (Table 1, first column).

**TABLE 1:** Concept extraction outcomes from the ASCEPT workshop, CC‐PEG and confirmatory survey

Concepts Extracted by Workshop groups (23, groups of 4–6)	Concepts extracted by Expert group (12)			Concepts from confirmatory survey (30)
1. Mechanism of drug action	Round one (extraction)	Round two (rating using criteria)	Round 3 (ranked most to least important)	Confirmatory survey
2. How the body handles a drug	1. Drug absorption	1. Drug absorption	1. Concentration response relationships	1. Concentration–response relationships 100%
3. Drug safety	2. Concentration response relationships	2. Concentration response relationships	2. Drug selectivity	2. Drug efficacy 100%
4. Medicines as therapeutics	3. Drugs and homeostasis	3. Drugs and homeostasis	3. Drug efficacy	3. Drug target 100%
5. Quantification of dose response	4. Drug excretion	4. Drug excretion	4. Drug affinity	4. Drug absorption 100%
6. Drug discovery & development	5. Drugs and complex systems	5. Drugs and complex systems	5. Mechanism of drug action	5. Drug tolerance 100%
7. Concentration response relationships	6. Drug distribution	6. Drug distribution	6. Drug target	6. Drug distribution 100%
8. Drug selectivity and specificity	7. Drug interactions	7. Drug safety/adverse drug reactions	7. Drug distribution	7. Individual variation 100%
9. Concentration occupancy relationships	8. Drug target	8. Drug target	8. Drug safety	8. Drug selectivity 96%
10. Sites of drug action	9. Drug metabolism	9. Drug metabolism	9. Drug metabolism	9. Bioavailability 96%
11. Absorption	10. Mechanism of drug action	10. Mechanism of drug action	10. Drug absorption	10. Drug safety 96%
12. Distribution	11. Drug efficacy	11. Drug efficacy	11. Individual variation	11. Drug potency 96%
13 Metabolism	12. Drug selectivity and specificity	12. Drug selectivity and specificity	12. Drugs and homeostasis	12. Drug metabolism 96%
14 Excretion	13. Drug affinity	13. Drug affinity	13. Drug excretion	13. Mechanism of drug action 92%
15. How are drugs handled by the body?	14. Statistical significance	14. Drug tolerance	14. Bioavailability	14. Therapeutic window 92%
16. Drug–receptor interactions	15. Individual variation	15. Individual variation	15. Drug tolerance	15. Drug excretion 92%
17. Drugs treating complex system		16. Bioavailability	16. Drugs and complex systems	16. Drug affinity 92%
18. Metabolism			17. Drug elimination	17. Drug elimination 91%
19. Dose matters			18. Therapeutic window	Below 80% agreement
20. Individual variation			19. Drug potency	18. Drugs and complex systems 79%
21. Drug efficacy				19. Drugs and homeostasis 76%
22. Selectivity				
23. ADME				
24. What the drug does to the body				
25. What the body does to the drug				
26. Drug–drug interactions				

The first column shows the concepts that the workshop groups extracted during the workshop, and the next three columns show the concepts that emerged from the three rounds of CC‐PEG extraction and refinement. The final column shows each concept from the confirmatory survey with the % agreement for inclusion of each concept as a core concept of pharmacology education.

### 2.7 Establishment of expert groups

At the completion of the workshop, 12 participants were chosen based on expressions of interest to form the core concepts in pharmacology expert group (CC‐PEG). The members had been pharmacology educators for an average of 17 years, had received an average of four teaching awards in pharmacology and had an average of 24 publications in pharmacology (a mix of educational research and biomedical research). Eleven of the 12 CC‐PEG members represent four of the six states and one of the two territories in Australia, with the twelfth representing New Zealand.

### 2.8 Expert group activities—extracting core concepts

CC‐PEG met virtually each fortnight for around 3 months.

#### 2.8.1 Round one

The first task for the group was to consolidate the 26 concepts that emerged from the group work during the workshop. Similar terms were consolidated to produce a list of 15 concepts for the group to work with.

#### 2.8.2 Round two

the working list of 15 concepts was then evaluated by each CC‐PEG member individually, using the criteria: *Is this concept useful to solve problems or predict outcomes?*; *Is this a big idea?*; *Is this an enduring idea?*; *Is this a topic or fact?*; *Can this idea be applied in multiple contexts?*; *Is this concept difficult for students?* The aggregate ratings were used to accept, reject, or flag a concept for further discussion, with an average of 80% agreement, via individual independent voting, that the concept met all criteria being used as the threshold for acceptance, and 50% as the threshold for further discussion.

#### 2.8.3 Round three

Each participant then indicated whether they *“believe each of the ideas below should be adopted as core concepts in pharmacology or not”*.

### 2.9 Final survey

A final survey (Data S2) of pharmacology educators from Australian and New Zealand was conducted in order to ensure that the core concepts that the CC‐PEG group had extracted from the earlier survey were still representative of the view of the wider group. Respondents were presented with each of the 18 concepts and asked to (i) evaluate the concepts using the criteria described earlier and (ii) agree or disagree with the inclusion of each concept as a core concept in pharmacology education. There were 30 respondents and 22 complete responses. Respondents taught (in order of frequency) medical, postgraduate research, biomedical science, science, pharmacy, nursing, and dentistry students, and 80% had PhDs as their highest earned academic degree. Almost half (48%) of respondents had been teaching for more than 20 years and 60% were male. Of note, the preponderance of males who teach medical students in the confirmatory survey was in contrast with the preponderance of females teaching science and biomedical science students in the exploratory survey.

### 2.10 Statistical analysis

A common question that arose during workshops and meetings was whether the background of the educator influenced their perception of core concepts in pharmacology. We tested this by comparing the ratings of each concept (shown in Table 2) between basic science and clinical educators as reported in the demographic item.

**TABLE 2:** Criterion‐based evaluation of the concepts by (i) CC‐PEG concept extraction (EG) and (ii) confirmatory survey participants (CS)

Concept	Useful to solve/predict		Big idea		Enduring idea		Applies to multiple contexts		Difficult for students	
	EG	CS	EG	CS	EG	CS	EG	CS	EG	CS
1. Drug absorption	90	76	70	44	90	64	100	80	70	12
2. Concentration response relationships	90	92	100	56	100	76	100	92	70	40
3. Drugs and homeostasis	70	68	90	52	100	52	100	64	90	48
4. Drug excretion	100	81	60	50	90	69	100	69	40	19
5. Drugs and complex systems	60	66	80	75	90	58	100	79	90	66
6. Drug distribution	80	84	70	52	90	64	100	76	70	48
7. Drug safety	100	76	100	72	100	64	100	84	80	40
8. Drug target	90	83	80	71	90	79	100	96	60	17
9. Drug metabolism	90	92	80	72	90	72	100	90	80	24
10. Mechanism of drug action	100	84	90	68	100	60	100	88	80	36
11. Drug efficacy	90	80	80	72	90	76	100	76	80	56
12. Drug selectivity	100	80	80	40	90	64	100	84	90	56
13. Drug affinity	100	67	70	50	90	75	100	75	70	29
14.Drug tolerance	100	72	70	48	90	52	100	76	90	60
15. Individual variation	100	80	90	76	100	56	100	72	80	52
16. Bioavailability	90	84	50	50	100	60	100	80	70	36
17. Drug potency	91	80	73	52	82	68	91	80	73	50
18. Therapeutic window	91	80	91	52	100	64	100	68	55	20
19. Drug elimination	100	91	91	61	100	61	91	70	91	26
Means	91	80	80	59	94	65	94	79	75	39

The data provided indicates the percentage of CC‐PEG members who agreed that the concept met each criterion. Ten of the 12 CC‐PEG members completed Round two, and 30 respondents completed the confirmatory survey.

*‘The discipline I primarily teach is’*. The ratings of core concepts by basic science educators were compared to those of clinical educators using a two‐way ANOVA, with one factor being educator discipline and the other being the core concepts criteria.

## 3 RESULTS

### 3.1 Data from British Pharmacological Society meeting: pilot survey

Nine respondents at the BPS meeting in 2019 were provided with very brief information about core concepts, and asked the following question via paper survey:

“Imagine your current/recent pharmacology students 3–5 years after their graduation. What few essential core concepts would you expect them to remember, understand deeply, and apply effectively in their professional work?”

Twenty‐nine terms were proposed as core concepts in the responses received, with 24 individual terms remaining, once identical terms or synonyms (e.g. “*mode of action”* and *“molecular mechanism of action”*) were combined. Most terms were used only by one respondent, whilst *efficacy*, *affinity*, and *mode of action* were used by at least two respondents. There were a number of terms proposed that were very specific (*“care in administering corticosteroids”*, *“asthma”*, *“calcium homeostasis”*). These findings indicated a need to provide criteria to guide respondents.

### 3.2 Survey data from ASCEPT meeting

Thirty‐three respondents at the ASCEPT workshop in 2020 were given information about core concepts, including the criteria described above, and asked the question shown above via online survey. One hundred and nine terms were used in the responses received. Of those 109 terms, many were identified by more than one respondent, either using identical terms or synonyms (e.g. “*concentration response relationship”* vs. “*relationship between concentration and response”*), such that there were 52 individual terms. The most common terms (including synonyms) were: *concentration–response relationships*; *mechanism of action of drug*; *efficacy*; *drug interactions*; *selectivity of action*; *potency*; *ADME*; *affinity*; *individual variation*; *drug discovery and development*; *drug safety*; *drug targets*; *homeostasis*; *therapeutic window*; *pharmacokinetics*. Terms used only by one respondent were more likely to be from disciplines that underpin pharmacology such as physics or biology (e.g. “*law of mass action”*, *“lipophilicity”*, *“competitive inhibition”*, *“systems physiology”*).

### 3.3 Workshop extraction

Twenty‐three workshop participants worked in groups of 4–6 online via Zoom break‐out rooms. They began by sharing their survey responses with each other. Each group then refined that list using the criteria as a guide to produce a maximum of 10 proposed core concepts to share with the wider group. A total of 39 proposed concepts was produced from all the groups. The breadth of concepts proposed by the groups varied from extremely broad (e.g. “*what the body does to the drug”)* to quite narrow (*“drug*‐*drug interactions”*). A working list of 26 individual concepts remained once common terms and synonyms were consolidated at the end of the workshop.

### 3.4 Extraction of core concepts by the CC‐PEG

#### 3.4.1 Round one

The CC‐PEG began with the 26 concepts extracted during the workshop and concluded with 15 concepts. This process involved consolidation of multiple terms that related to a common central concept—for example “*concentration*‐*response relationships”* was chosen to represent terms such as “*quantification of the dose”* and “*dose matters”*.

#### 3.4.2 Round two (see Table 1)

Group members then rated each of the 15 proposed concepts using the five criteria we developed and listed any concepts they believed to be missing. *Drug tolerance* and *drug bioavailability* were added to the list during this process. All but one concept was rated by at least 80% of CC‐PEG members as meeting the five criteria and were progressed to the next stage. *Drug tolerance* did not meet the 80% agreement threshold, but was flagged for further discussion, given that the percentage agreement across the six criteria for this concept was very close to the threshold. One concept met the 80% agreement threshold but was deemed to be out of scope (*“statistical significance”*), leaving 16 remaining concepts.

#### 3.4.3 Round three (see Table 2)

Each participant then indicated whether they believed *“whether each of the ideas below should be adopted as core concepts in pharmacology or not”* and indicated their order of importance from 1 (most important) to 16 (least important). Three concepts (*drugs and homeostasis*, *drug tolerance*, and *drugs and complex systems*) did not meet the 80% agreement threshold, but were viewed as having sufficient merit to be discussed further. In the ensuing discussion, once the group had seen and discussed the approved list, three further concepts were added: *drug elimination*, *therapeutic window*, and *drug potency*. The group agreed that the confirmatory survey would include these additional concepts, resulting in a list of 19 concepts.

### 3.5 Confirmatory survey

Twenty‐five pharmacology educators responded to the final survey regarding the 19 proposed core concepts produced by the CC‐PEG, providing 22 complete responses. Seventeen of the 19 concepts reached the pre‐determined threshold for endorsement of the concepts—80% of respondents agreeing to statement *“this concept should be included as a core concept of pharmacology education”*. Two concepts did not reach the 80% agreement threshold: *drugs and homeostasis* and *drugs and complex systems*. The final column of Table 2 shows the percent agreement for the inclusion of each concept.

### 3.6 Retrospective analysis of survey items

The responses of the BPS pilot survey and ASCEPT survey were analysed to determine which of the 19 core concepts in pharmacology produced in the study were present in the earlier survey responses. Of the 24 terms used by BPS survey respondents, five were later determined to be core concepts of pharmacology education. Of the 55 unique terms used by ASCEPT survey respondents, 15 were later determined to be core concepts.

### 3.7 Basic science versus clinical educators

**FIGURE 2:**
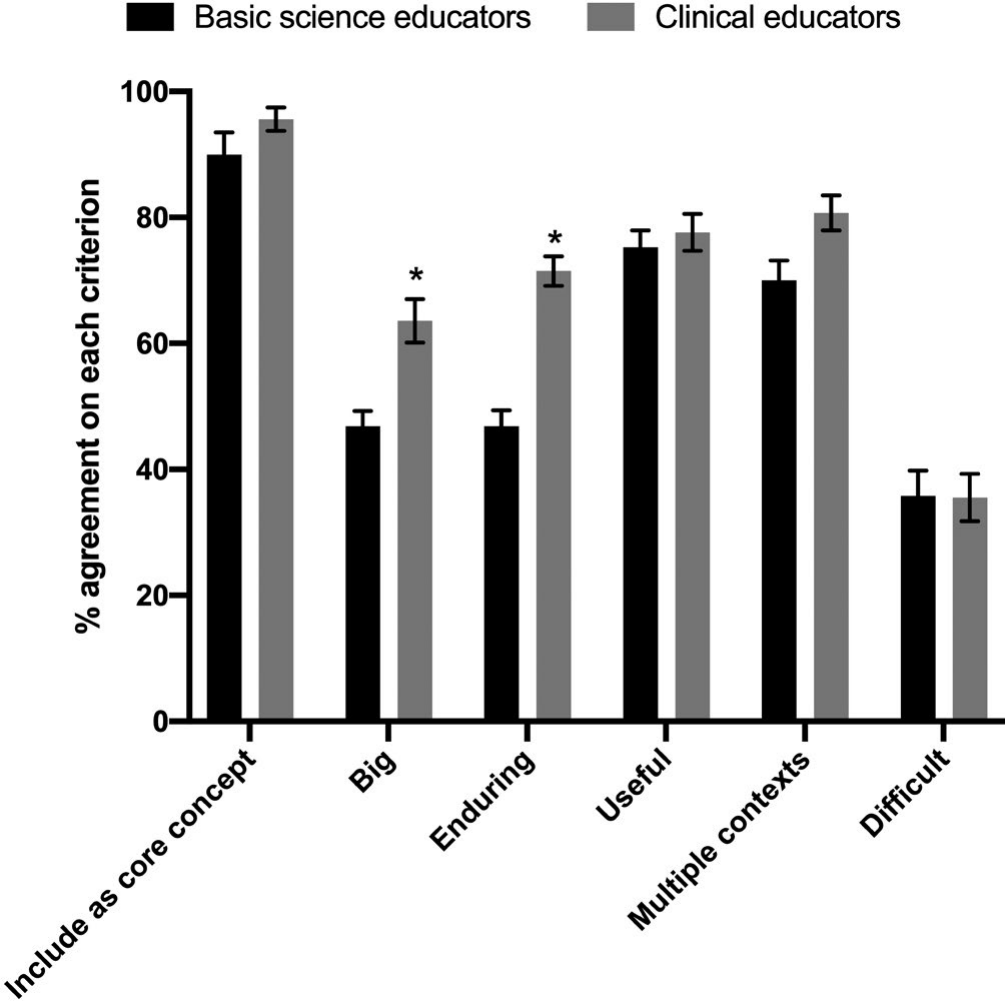
Ratings of core concepts by basic science and clinical educators using five criteria. Data are percent agreement for each group of educators for all 19 proposed concepts combined. * indicate a significant difference from the basic science educator agreement (ANOVA, *p* < .05)

We tested whether the background of the educator influenced their perception of core concepts in pharmacology by comparing the ratings of each concept (Table 2) between basic science and clinical educators. Overall, 10 basic science educators gave lower ratings than their 12 clinical educator counterparts (Figure 2, ANOVA, *F* (1, 216) = 32.8, *p* < .0001).

### 3.8 Organization of core concepts

**FIGURE 3:**
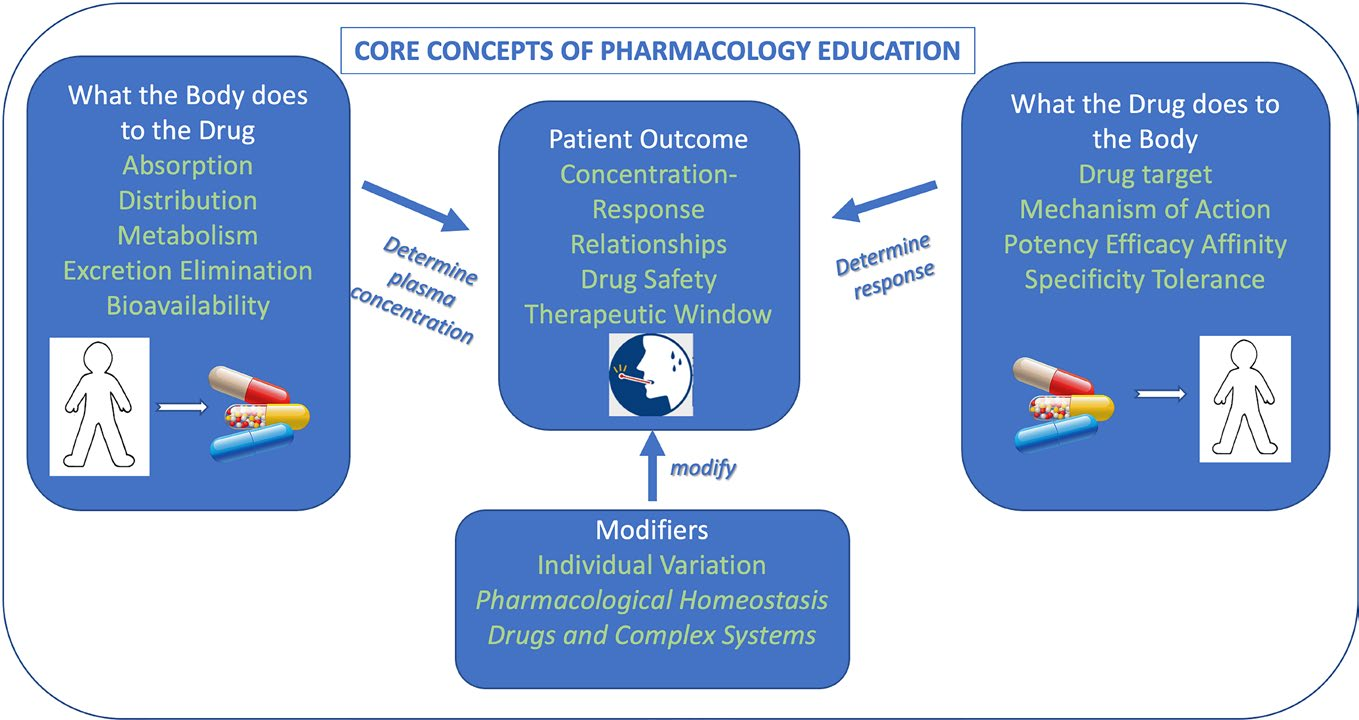
A diagrammatic representation of the final list of concepts produced by the CC‐PEG

During group discussions, it was clear that the core concepts naturally fell into three distinct sub‐groups. Figure 3 provides a diagrammatic representation of the final list of concepts produced by the CC‐PEG.

## 4 DISCUSSION

We developed a rigorous method of identification of core concepts using a Delphi approach combined with Expert Group analysis, using novel criteria that we developed and refined after several rounds of trial and error. Starting with 109 terms proposed in the ASCEPT survey, workshop groups produced a starting list of 39 terms and refined this to a list of 26. The CC‐PEG worked together to consolidate this list to 15 concepts using the five criteria, and further discussion resulted in 19 proposed core concepts that pharmacology students should be expected to know and apply years after graduation. A confirmatory survey of ASCEPT pharmacologists revealed greater than 80% agreement for 17 of 19 proposed concepts, with the remaining two concepts gaining just under 80% agreement. The percentage agreement of the final list of concepts by a broad group of pharmacology educators provides evidence that our novel methodology resulted in a list of concepts that were strongly endorsed by experts who had not been involved in the process.

### 4.1 The need for criteria and expert groups

When educators were asked in this study to name core concepts in pharmacology education, two general principles emerged: (i) some guidance was necessary as to the definition of core concepts to avoid a large number of proposed terms and (ii) survey responses, even from participants who have read and understood the criteria, require an additional analytical process to extract concepts from the inevitable *“word salad”* ^16^ that is produced.

### 4.2 Novel criteria for core concepts

We found that guidance, in the form of evaluation criteria, was necessary to support educators to understand the question that was being asked of them. Our criteria used definitions that were reported in previous studies, but not used in those studies to judge whether candidate concepts should be adopted. Additionally, we unpacked the critical elements of what was “*important to know*”. *Big ideas*,^32^ coupled with “*applicable in multiple contexts”* ^26^ were useful in rejecting terms that were too specific to be core concepts. *Enduring* ^17^, ^18^, ^35^ and *difficult* for students to understand^17^ provided reference to the history of the discipline and the reality of teaching students, respectively, and *“having utility to explain*/*predict outcomes”* ^14^ provided a way to ensure that the concepts were useful for students and also assessable by educators.

Individual backgrounds and biases were more heavily represented in the pilot survey, prior to the explicit use of criteria. For example, one respondent who reported teaching “cardio and neuro” proposed calcium homeostasis as a core concept. Responses to the ASCEPT exploratory survey, in which the core concepts were explicitly explained to participants, were much less frequently specific to a particular topic within the discipline. Hence, when the CC‐PEG analysed the 19 core concepts that survived multiple rounds of analysis using the criterion *Big idea*, there was strong agreement (79% across all concepts). Similarly, there was strong agreement by the final stage of CC‐PEG evaluation that the 19 concepts *useful* and *enduring*, and all concepts were seen as *applying to multiple contexts*. The criterion *difficult for students* showed the greatest variance, which is perhaps as expected given the variety of student cohorts and teaching contexts involved.

### 4.3 Expert groups

The use of criteria alone in survey format was insufficient to produce a usable list of core concepts. Our workshop was a quick and effective way to narrow down a longer list of 55 unique terms from the exploratory survey to a manageable list of 26 terms. Some of this work was relatively straightforward; for example, we consolidated terms such as “*quantification of the dose”* and “*dose matters”* into a single concept called “*concentration*‐*response relationships”*. However, other matters were more complex, including the CC‐PEG discussions into which of the proposed concepts regarding drug efficacy, agonists and antagonists should be included, which stretched across many meetings.

The expert groups synthesized ideas and consolidated terms from the exploratory survey. For example, “*homeostasis”* was referred to in both pilot and exploratory survey stages. This concept is already established as a core concept of physiology,^14^ and therefore, the CC‐PEG explored whether there was a related concept that applied to pharmacology. The group soon established that drug action is both moderated by homeostatic processes (e.g. blunting of anti‐hypertensive drug response) and in turn affects homeostatic set points (e.g. re‐setting of blood pressure set point in patients receiving these drugs). Hence, “*drugs and homeostasis”* was proposed as a *latent* core concept, inferred from survey responses in pharmacology, analogous to latent variables in psychometric testing.^39^ The confirmatory survey endorsement for this concept was just below the threshold we set for acceptance, indicating perhaps that a better name is required for this newly synthesized concept: we will investigate the term “*pharmacological homeostasis”* in future studies.

Most (15) of the concepts that were adopted in the final list of core concepts were present in some form in the exploratory survey, and 17 of 19 were endorsed by greater than 80% of respondents to the confirmatory survey conducted at the end of the study, with the two remaining concepts gaining majority (over 70%) support. These outcomes provide confidence that the core concepts in our list are indicative of the views of Australian and New Zealand pharmacology educators. This view is supported by a comment from a participant in the confirmatory survey, who, in response to the prompt *“Please add any final comments or suggestions as to how we can improve this list of core concepts of pharmacology education”* responded with *“Any that I can think of seem to be derivative of one or more of the core concepts from the draft list”*. We note that we are currently working on the unpacking of each concept into sub‐concepts to increase the usefulness of the core concepts list to educators.

### 4.4 Comparison of our core concepts with other resources

Our final list of core concepts in pharmacology education contains a number of terms that are also present in existing pharmacology resources online. The IUPHAR Pharmacology Education Project (https://www.pharmacologyeducation.org/pharmacology) list of topics includes *drug absorption*, *distribution*, *metabolism*, *and excretion*, as well as synonyms or related terms for *tolerance* (desensitization and tachyphylaxis), *drug targets* (receptors, enzymes ion channels), *drug interactions* and *individual variation*. The British Pharmacological Society Core Knowledge curriculum, (https://www.bps.ac.uk/education‐engagement/teaching‐pharmacology/undergraduate‐curriculum‐(1)/undergraduate‐curriculum) contains a section on Therapeutic Principles of Drug Action, which includes the words *target*, *drug absorption*, *distribution*, *metabolism*, and *excretion*, and other topics that relate to our core concepts. Additionally, the BPS curriculum core knowledge statements refer to *principles of drug action*, drug targets, *drug absorption*, *distribution*, *metabolism*, and *excretion*, *safety pharmacology and how physiological and pathophysiological processes are affected by drug action*. Finally, the Aquifer Sciences Pharmacology Core Concepts list (https://www.aquifersciences.org/concepts?q%5Bsd%5D=Pharmacology ) includes the terms *absorption*, *distribution*, *metabolism* and *excretion*, *drug interactions*, *mechanism of action*, and references to sources of *individual variation*.

The significant overlap of core concepts produced in this study with terms used in online pharmacology curricular resources indicates that our methodology “*covers the ground”*. Our criterion‐based method involving exploratory and confirmatory survey on either side of expert group extraction provides confidence that our list are indeed core concepts of pharmacology education.

### 4.5 Limitations

The study was conducted in Australia and involved in total around 50 educators from Australia and New Zealand, which is only a small subset of pharmacology educators worldwide. However, as described, there is substantial agreement in subject matter with published pharmacology knowledge curricula, suggesting that these concepts are relevant to the broader pharmacology education community. Now that we have established the criteria and methodology, we are currently conducting the same series of research phases with pharmacology educators globally We also note that the scope of our work does not cover concepts specific to particular organ systems or disease states, such as cardiovascular or central nervous system pharmacotherapeutics.

The demographic disparity between the exploratory survey participants (and CC‐PEG members) on the one hand and the confirmatory survey participants on the other is noteworthy and represents both a strength and limitation of the study. The exploratory survey participants and CC‐PEG members were predominantly women teaching science and biomedical science students with a significant minority being men who teach medical and health sciences students. This was the reverse of the majority male group who teach medical and other health professions students in the confirmatory survey. One likely explanation of the gender disparity relates to the recruitment of participants—the exploratory survey was held within a (predominantly female) ASCEPT education section workshop, whilst the confirmatory survey was distributed by the Secretariat of the Society, which has a predominantly male composition.

The strong agreement with the proposed core concepts observed in the confirmatory survey indicates that clinician educators and basic science educators, and male and female educators have highly consistent views about the core concepts of pharmacology education. On the other hand, ideally the exploratory and confirmatory surveys would have been completed by similar groups. On balance, each stage has representation from men and women, as well as both science and health professions educators.

### 4.6 Future directions

This list of core concepts in pharmacology education should be tested and validated beyond Australia and New Zealand. A parallel project to use data mining of core concepts from pharmacology textbooks is underway, and can be used to validate and refine this list.

## DISCLOSURE

The authors have no conflicts of interest with respect to this study

## Author contributions

Made substantial contributions to conception and design, or acquisition of data, or analysis and interpretation of data; and Been involved in drafting the manuscript or revising it critically for important intellectual content; and Given final approval of the version to be published. Each author should have participated sufficiently in the work to take public responsibility for appropriate portions of the content; and Agreed to be accountable for all aspects of the work in ensuring that questions related to the accuracy or integrity of any part of the work are appropriately investigated and resolved.

### Open Research Badges

This article has earned Open Data, Open Materials and Preregistered Research Design badges. Data, materials and the preregistered design and analysis plan are available in the article.

## Supporting information

Data S1

Data S2

## Data Availability

The data that support the findings of this study are available from the corresponding author upon reasonable request.
