# Supplementary material for: Identifying the core concepts of pharmacology education
Source: Pharmacol Res Perspect. 2021 Jul 21;9(4):e00836. doi: 10.1002/prp2.836 (PMC8292785; doi:10.1002/prp2.836)
Supplement: Supplementary file 1 — Data S1 [file PRP2-9-e00836-s002.docx]

Core Concepts - pharmacology - V2

Start of Block: Introduction

Defining the **core concepts** that underpin pharmacology education  A new paradigm for pharmacology education   ASCEPT core concepts in pharmacology expert group Paul White, Elizabeth Davis, Marina Junqueira Santiago, Lynette Fernandez, Anna-Marie Babey, Alison.Shield, Barbara Kemp-Harper, Gregg Maynard, Hesham Al-Sallami, Ian Musgrave, Suong Ngoc Thi Ngo, Tina Hinton and Tom Angelo.

Q27

The core concepts in pharmacology expert group have been working within the education forum of ASCEPT to determine what those who teach pharmacology think are the essential core concepts – the fundamental big ideas – pharmacology students must master prior to graduation. We invite you to share your expertise by providing your opinion, using criteria that we provide, of each of the 19 core concepts that the team have identified using the input from ASCEPT members. The survey will take approximately 15 minutes to complete.

Do you want to move straight to the survey questions about core concepts?

- Yes (1)
- No, I want to understand more about core concepts first (2)

**What are ‘core concepts’ and why do they matter? A bit of background**

 Core concepts are those big ideas that experts in a domain or discipline agree are fundamental, enduring and useful and, therefore, essential for students to learn, understand, remember, and apply. Core concepts are not facts, topics or information. Nobel Prize-winning physicist and educator Carl Weiman, PhD, defines scientific concepts as ideas “… that can be applied in multiple contexts to explain and/or predict outcomes.” Core concepts are the most critical, powerful and useful ideas, often providing a discipline’s foundation and structure.    **Characteristics of core concepts** We have developed the following criteria based on the work of Joel Michael’s group in physiology, ideas from physicist Carl Wieman, and feedback from the PharmAlliance Education Domain. We assert that core concepts are: Applicable across multiple contexts Big ideas. Critical, powerful and useful ideas, often a key part of a discipline’s conceptual framework and structure.  Useful - can be applied in multiple contexts to explain and/or predict outcomes (have utility) Enduring. Lasting – unlikely to change over a generation Difficult for students to master For example, pharmacokinetics is a topic within pharmacology – whilst there are core concepts related to PK, it is itself a topic rather than a core concept. The existence of beta 1 adrenoceptors is a fact rather than a concept. “Drug potency” is likely to be a core concept in pharmacology, to use a non-controversial example.   Knowledge in all pharmacology-related disciplines is constantly changing and growing – often exponentially. By contrast, available time and resources for teaching and learning are constant or shrinking. Since we cannot teach everything, knowing which are the essential core concepts allows educators to more effectively and efficiently focus and align their curricula, teaching, and assessment.

Examples of core concepts in other disciplines     Statistics   * Confidence   * Correlation   * Error   * Validity   Physiology   * Homeostasis   * Feedback loops   * Cell:cell communication   Based on their lists of core concepts, these disciplines, among others, have developed powerful assessment tools known as **concept inventories**. Concept inventories assess students’ deep understanding – not simply memorization – of core concepts. They can be used pre- and post-instruction, by individuals or programs. Concept inventories can also be used to identify students’ common preconceptions or misconceptions. Graduates of pharmacology courses and subjects require conceptual knowledge from this disciplines to perform the many roles related to the development and/or safe and effective use of medicines. This survey is a first step toward constructing an evidence-based consensus list or lists of the core concepts that form that foundational knowledge. Thank you in advance for your help in developing this consensus list and the concept inventories that will follow.

Human Ethics. MUHREC project ID 22727 “Core concepts” has been approved as low risk by the Monash University Human Research Ethics Committee. Your participation in the survey and workshop implies consent for your de-identified responses to be used by the research team for the purposes of the project.

End of Block: Introduction

Start of Block: Second Response block

Background information about you
To better understand and contextualize your responses, we request your responses to the following five questions:
The students I teach are [choose all that apply]:

- Dentistry students (3)
- Nursing students (1)
- Medical students (9)
- Osteopathy students (11)
- Pharmacy students (2)
- Physiotherapy students (10)
- Science students (4)
- Biomedical science students (12)
- All post-graduate research (Masters / PhD) students (5)
- Not applicable (I don't teach) (8)
- Other (7) ________________________________________________

The discipline I primarily teach is (please select one):

- Basic pharmacology (1)
- Systems pharmacology / therapeutics (15)
- Clinical pharmacology (16)
- Microbiology / anti-infectives (2)
- ADME / pharmacokinetics (4)
- Drug formulation / compounding (5)
- Drug development / regulation (6)
- Medicinal chemistry (8)
- Pharmacotherapy / clinical management (10)
- Physiology (11)
- Epidemiology / statistics (12)
- Other (please describe) (13) ________________________________________________

Typical enrolments in each cohort that I teach are:

- <50 (1)
- 51-100 (4)
- 101-150 (5)
- 151-200 (6)
- 201-250 (7)
- >250 (3)

My highest earned academic degree is:

- PhD (2)
- MBBS (4)
- Master (5)
- Bachelor (6)
- Other: please enter your degree (7) ________________________________________________

I have been teaching pharmacology for:

- Less than 3 years (1)
- 3-5 years (2)
- 6-10 years (3)
- 11-20 years (4)
- >20 years (5)

I identify as:

- Male (1)
- Female (2)
- Prefer not to respond (5)
- Other (4) ________________________________________________

| Page Break |  |
| --- | --- |

Q48 We would now like to get your view on the 19 concepts that have been proposed by the ASCEPT education core concepts team.  For each concept, please read the definition and then consider whether i) the concept meets any or all of the five criteria and ii) choose ONE of the final TWO options to indicate whether in your view the concept should be included as a core concept for pharmacology education.

Q28 Concept 1: The concept ***'Drug Absorption'***refers to the movement of the drug from its site of administration. Please choose any of the following that you believe are accurate statements about this concept. Please choose ALL OF THE FIRST FIVE OPTIONS THAT APPLY, and then one of the FINAL TWO options.

- It is useful to solve problems or predict outcomes (1)
- It is an enduring idea (2)
- It can be applied to multiple contexts (3)
- It is difficult for students to attain (4)
- It is a big idea (7)
- It SHOULD be included as a core concept of pharmacology education (5)
- It SHOULD NOT not be included as a core concept of pharmacology education (6)

Q29 Concept 2: The concept '***Drug Distribution'*** refers to the movement of the drug around the body. Please choose any of the following that you believe are accurate statements about this concept. Please choose ALL OF THE FIRST FIVE OPTIONS THAT APPLY, and then one of the FINAL TWO options.

- It is useful to solve problems or predict outcomes (1)
- It is an enduring idea (2)
- It can be applied to multiple contexts (3)
- It is difficult for students to attain (4)
- It is a big idea (7)
- It SHOULD be included as a core concept of pharmacology education (5)
- It SHOULD NOT not be included as a core concept of pharmacology education (6)

Q32 Concept 3:  The concept ***'Drug metabolism'*** refers to the biotransformation of the drug. Please choose any of the following that you believe are accurate statements about this concept. Please choose ALL OF THE FIRST FIVE OPTIONS THAT APPLY, and then one of the FINAL TWO options.

- It is useful to solve problems or predict outcomes (1)
- It is an enduring idea (2)
- It can be applied to multiple contexts (3)
- It is difficult for students to attain (4)
- It is a big idea (7)
- It SHOULD be included as a core concept of pharmacology education (5)
- It SHOULD NOT not be included as a core concept of pharmacology education (6)

Q31 Concept 4: The concept ***'Drug excretion'*** refers to the removal of drug from the body. Please choose any of the following that you believe are accurate statements about this concept. Please choose ALL OF THE FIRST FIVE OPTIONS THAT APPLY, and then one of the FINAL TWO options.

- It is useful to solve problems or predict outcomes (1)
- It is an enduring idea (2)
- It can be applied to multiple contexts (3)
- It is difficult for students to attain (4)
- It is a big idea (7)
- It SHOULD be included as a core concept of pharmacology education (5)
- It SHOULD NOT not be included as a core concept of pharmacology education (6)

Q30 Concept 5:  The concept ***'Drug elimination'*** refers to the irreversible removal of the drug and its metabolites from the body through a combination of metabolic and excretory processes. Please choose ALL of the following that you believe are accurate statements about this concept. Please choose ANY OF THE FIRST FIVE OPTIONS THAT APPLY, and then one of the FINAL TWO options.

- It is useful to solve problems or predict outcomes (1)
- It is an enduring idea (2)
- It can be applied to multiple contexts (3)
- It is difficult for students to attain (4)
- It is a big idea (7)
- It SHOULD be included as a core concept of pharmacology education (5)
- It SHOULD NOT not be included as a core concept of pharmacology education (6)

Q33 Concept 6:  The concept ***'Bioavailability'*** refers to the fraction of a drug dose which reaches the systemic circulation as intact drug. Please choose any of the following that you believe are accurate statements about this concept. Please choose ALL OF THE FIRST FIVE OPTIONS THAT APPLY, and then one of the FINAL TWO options.

- It is useful to solve problems or predict outcomes (1)
- It is an enduring idea (2)
- It can be applied to multiple contexts (3)
- It is difficult for students to attain (4)
- It is a big idea (7)
- It SHOULD be included as a core concept of pharmacology education (5)
- It SHOULD NOT not be included as a core concept of pharmacology education (6)

Q49 Concept 7: The concept ***‘Drug Potency’*** refers to an index of the concentration required for a given effect. Please choose any of the following that you believe are accurate statements about this concept. Please choose ALL OF THE FIRST FIVE OPTIONS THAT APPLY, and then one of the FINAL TWO options.

- It is useful to solve problems or predict outcomes (1)
- It is an enduring idea (2)
- It can be applied to multiple contexts (3)
- It is difficult for students to attain (4)
- It is a big idea (7)
- It SHOULD be included as a core concept of pharmacology education (5)
- It SHOULD NOT not be included as a core concept of pharmacology education (6)

Q34 Concept 8: The concept ***'Therapeutic Window'*** refers to the range between a lower boundary concentration at which clinical efficacy is lost and an upper boundary defined by the appearance of drug related toxicity. Please choose any of the following that you believe are accurate statements about this concept. Please choose ANY OF THE FIRST FIVE OPTIONS THAT APPLY, and then one of the FINAL TWO options.

- It is useful to solve problems or predict outcomes (1)
- It is an enduring idea (2)
- It can be applied to multiple contexts (3)
- It is difficult for students to attain (4)
- It is a big idea (7)
- It SHOULD be included as a core concept of pharmacology education (5)
- It SHOULD NOT not be included as a core concept of pharmacology education (6)

Q35 Concept 9:  The concept ***‘Drug Target’*** refers to the site where the drug binds to produce a response. Please choose any of the following that you believe are accurate statements about this concept. Please choose ALL OF THE FIRST FIVE OPTIONS THAT APPLY, and then one of the FINAL TWO options.

- It is useful to solve problems or predict outcomes (1)
- It is an enduring idea (2)
- It can be applied to multiple contexts (3)
- It is difficult for students to attain (4)
- It is a big idea (7)
- It SHOULD be included as a core concept of pharmacology education (5)
- It SHOULD NOT not be included as a core concept of pharmacology education (6)

Q36 Concept 10: The concept ***‘Mechanism of Drug Action’*** refers to the way in which a drug interacts with its target to modify cell function. Please choose any of the following that you believe are accurate statements about this concept. Please choose ALL OF THE FIRST FIVE OPTIONS THAT APPLY, and then one of the FINAL TWO options.

- It is useful to solve problems or predict outcomes (1)
- It is an enduring idea (2)
- It can be applied to multiple contexts (3)
- It is difficult for students to attain (4)
- It is a big idea (7)
- It SHOULD be included as a core concept of pharmacology education (5)
- It SHOULD NOT not be included as a core concept of pharmacology education (6)

Q37 Concept 11:  The concept ***‘Drug Efficacy’*** refers to the ability of a drug to produce the desired response. Please choose any of the following that you believe are accurate statements about this concept. Please choose ALL OF THE FIRST FIVE OPTIONS THAT APPLY, and then one of the FINAL TWO options.

- It is useful to solve problems or predict outcomes (1)
- It is an enduring idea (2)
- It can be applied to multiple contexts (3)
- It is difficult for students to attain (4)
- It is a big idea (7)
- It SHOULD be included as a core concept of pharmacology education (5)
- It SHOULD NOT not be included as a core concept of pharmacology education (6)

Q38 Concept 12:   The concept ***‘Drug Selectivity’*** refers to the concentration-dependent preference of a drug for one target over others. Please choose any of the following that you believe are accurate statements about this concept. Please choose ALL OF THE FIRST FIVE OPTIONS THAT APPLY, and then one of the FINAL TWO options.

- It is useful to solve problems or predict outcomes (1)
- It is an enduring idea (2)
- It can be applied to multiple contexts (3)
- It is difficult for students to attain (4)
- It is a big idea (7)
- It SHOULD be included as a core concept of pharmacology education (5)
- It SHOULD NOT not be included as a core concept of pharmacology education (6)

Q39 Concept 13:  The concept ‘***Drug Affinity’*** refers to the ability of a drug to bind to its target. Please choose any of the following that you believe are accurate statements about this concept. Please choose ALL OF THE FIRST FIVE OPTIONS THAT APPLY, and then one of the FINAL TWO options.

- It is useful to solve problems or predict outcomes (1)
- It is an enduring idea (2)
- It can be applied to multiple contexts (3)
- It is difficult for students to attain (4)
- It is a big idea (7)
- It SHOULD be included as a core concept of pharmacology education (5)
- It SHOULD NOT not be included as a core concept of pharmacology education (6)

Q40 Concept 14:.  The concept ***‘Concentration Response Relationships’*** refers to the relationship between increasing drug concentration and magnitude of response. Please choose ALL of the following that you believe are accurate statements about this concept. Please choose ANY OF THE FIRST FIVE OPTIONS THAT APPLY, and then one of the FINAL TWO options.

- It is useful to solve problems or predict outcomes (1)
- It is an enduring idea (2)
- It can be applied to multiple contexts (3)
- It is difficult for students to attain (4)
- It is a big idea (7)
- It SHOULD be included as a core concept of pharmacology education (5)
- It SHOULD NOT not be included as a core concept of pharmacology education (6)

Q41 Concept 15:  The concept ‘***Drug Safety’*** refers to the balance of therapeutic benefits against harmful effects. Please choose any of the following that you believe are accurate statements about this concept. Please choose ALL OF THE FIRST FIVE OPTIONS THAT APPLY, and then one of the FINAL TWO options.

- It is useful to solve problems or predict outcomes (1)
- It is an enduring idea (2)
- It can be applied to multiple contexts (3)
- It is difficult for students to attain (4)
- It is a big idea (7)
- It SHOULD be included as a core concept of pharmacology education (5)
- It SHOULD NOT not be included as a core concept of pharmacology education (6)

Q42 Concept 16:   The concept ***‘Drug Tolerance’*** refers to the reduced response to a drug following repeated or prolonged exposure. Please choose any of the following that you believe are accurate statements about this concept. Please choose ALL OF THE FIRST FIVE OPTIONS THAT APPLY, and then one of the FINAL TWO options.

- It is useful to solve problems or predict outcomes (1)
- It is an enduring idea (2)
- It can be applied to multiple contexts (3)
- It is difficult for students to attain (4)
- It is a big idea (7)
- It SHOULD be included as a core concept of pharmacology education (5)
- It SHOULD NOT not be included as a core concept of pharmacology education (6)

Q43 Concept 17:  The concept ***'Drugs and Homeostasis'*** refers to the important interplay between drug response and body homeostasis. Please choose any of the following that you believe are accurate statements about this concept. Please choose ALL OF THE FIRST FIVE OPTIONS THAT APPLY, and then one of the FINAL TWO options.

- It is useful to solve problems or predict outcomes (1)
- It is an enduring idea (2)
- It can be applied to multiple contexts (3)
- It is difficult for students to attain (4)
- It is a big idea (7)
- It SHOULD be included as a core concept of pharmacology education (5)
- It SHOULD NOT not be included as a core concept of pharmacology education (6)

Q44 Concept 18:  The concept ***‘Drugs and complex systems’*** describes the important interplay between drugs and patients as a whole integrated body system comprised of cells, organs and systems. Please choose ALL of the following that you believe are accurate statements about this concept. Please choose ANY OF THE FIRST FIVE OPTIONS THAT APPLY, and then one of the FINAL TWO options.

- It is useful to solve problems or predict outcomes (1)
- It is an enduring idea (2)
- It can be applied to multiple contexts (3)
- It is difficult for students to attain (4)
- It is a big idea (7)
- It SHOULD be included as a core concept of pharmacology education (5)
- It SHOULD NOT not be included as a core concept of pharmacology education (6)

Q45 Concept 19:  The concept ***‘Individual Variation’*** describes the fact that individuals respond differently to a given drug, due to exogenous and endogenous factors that influence drug availability and/or action. Please choose ALL of the following that you believe are accurate statements about this concept. Please choose ANY OF THE FIRST FIVE OPTIONS THAT APPLY, and then one of the FINAL TWO options.

- It is useful to solve problems or predict outcomes (1)
- It is an enduring idea (2)
- It can be applied to multiple contexts (3)
- It is difficult for students to attain (4)
- It is a big idea (7)
- It SHOULD be included as a core concept of pharmacology education (5)
- It SHOULD NOT not be included as a core concept of pharmacology education (6)

Q46 Now that you have seen the draft list of core concepts of pharmacology education, please name any concepts that you think are MISSING from the list.

________________________________________________________________

Q50 Please add any final comments or suggestions as to how we can improve this list of core concepts of pharmacology education.

________________________________________________________________

 Only aggregate summaries of this survey data will ever be reported and published. Any and all individual data will be de-identified prior to analysis.
That said, if you would like to receive reports on this core concepts research project, please feel free to insert your email address below.

________________________________________________________________

End of Block: Second Response block
