# Supplementary material for: Identifying the core concepts of pharmacology education
Source: Pharmacol Res Perspect. 2021 Jul 21;9(4):e00836. doi: 10.1002/prp2.836 (PMC8292785; doi:10.1002/prp2.836)
Supplement: Supplementary file 2 — Data S2 [file PRP2-9-e00836-s001.docx]

Core Concepts - pharmacology

Start of Block: Introduction

Defining the Core Science Concepts that underpin pharmacology education  A New Paradigm for pharmacology Education   Paul White, Tom Angelo, Jacqui McLaughlin, Mike Munday, Elizabeth DavisOn behalf of the Education Domain of PharmAllianceIn conjunction with ASCEPT

Q27

This survey aims to determine what those who teach in schools and colleges that teach pharmacology think are the essential core concepts – the fundamental big ideas – pharmacology students must master prior to graduation. We invite you to share your expertise by responding and, in turn, we will share initial summarized findings with you and all other respondents. The survey will take approximately 15 minutes to complete.

Do you want to move straight to the survey questions about core concepts?

- Yes (1)
- No, I want to understand more about core concepts first (2)

What are ‘core concepts’ and why do they matter? A bit of backgroundCore concepts are those big ideas which experts in a domain or discipline agree are fundamental, enduring and useful and, therefore, essential for students to learn, understand, remember, and apply. Core concepts are not facts, topics or information. Nobel Prize-winning physicist and educator Carl Weiman, PhD, defines scientific concepts as ideas “… that can be applied in multiple contexts to explain and/or predict outcomes.” Core concepts are the most critical, powerful and useful ideas, often providing a discipline’s foundation and structure. Characteristics of core conceptsWe have developed the following criteria based on the work of Joel Micheal’s group in physiology, ideas from physicist Carl Wieman, and feedback from the PharmAlliance Education DomainWe assert that core concepts are:•       Applicable across multiple contexts•       Big ideas. Critical, powerful and useful ideas, often a key part of a discipline’s conceptual framework and structure. •       Useful - can be applied in multiple contexts to explain and/or predict outcomes (have utility)•       Enduring. Lasting – unlikely to change over a generation•       Not a topic or a fact•       For example, pharmacokinetics is a topic within pharmacology – whilst there are core concepts related to PK, it is itself a topic rather than a core concept. The existence of b1 adrenoceptors is a fact rather than a concept. “Drug potency” is likely to be a core concept in pharmacology, to use a non-controversial example  Knowledge in all pharmacology-related disciplines is constantly changing and growing – often exponentially. By contrast, available time and resources for teaching and learning are constant or shrinking. Since we cannot teach everything, knowing which are the essential core concepts allows educators to more effectively and efficiently focus and align their curricula, teaching, and assessment.

Examples of core concepts in other disciplines     Statistics   * Confidence   * Correlation   * Error   * Validity   Physiology   * Homeostasis   * Feedback loops   * Cell:cell communication   Based on their lists of core concepts, these disciplines, among others, have developed powerful assessment tools known as **concept inventories**. Concept inventories assess students’ deep understanding – not simply memorization – of core concepts. They can be used pre- and post-instruction, by individuals or programs. Concept inventories can also be used to identify students’ common preconceptions or misconceptions. Graduates of pharmacology courses and subjects require conceptual knowledge from this disciplines to perform the many roles related to the development and/or safe and effective use of medicines. This survey is a first step toward constructing an evidence-based consensus list or lists of the core concepts that form that foundational knowledge. Thank you in advance for your help in developing this consensus list(s) and the concept inventories that will follow.

Human Ethics. MUHREC project ID 22727 “Core concepts” has been approved as low risk by the Monash University Human Research Ethics Committee. Your participation in the survey and workshop implies consent for your deidentified responses to be used by the research team for the purposes of the project.

End of Block: Introduction

Start of Block: Second Response block

Background information about you
To better understand and contextualize your responses, we request your responses to the following five questions:
The students I teach are [choose the most appropriate response below]

- Dentistry students (3)
- Nursing students (1)
- Medical Students (9)
- Osteopathy students (11)
- Pharmacy students (2)
- Physiotherapy students (10)
- Science students (4)
- Biomedical science students (12)
- All post-graduate research (Masters / PhD) students (5)
- Not applicable (I don't teach) (8)
- Other (7) ________________________________________________

The discipline I primarily teach is (please select one)

- Basic pharmacology (1)
- Systems pharmacology / therapeutics (15)
- Clinical pharmacology (16)
- Microbiology / anti-infectives (2)
- ADME / pharmacokinetics (4)
- Drug Formulation / compounding (5)
- Drug development / regulation (6)
- Medicinal chemistry (8)
- Pharmacotherapy / clinical management (10)
- Physiology (11)
- Epidemiology / statistics (12)
- Other (please describe) (13) ________________________________________________

Typical enrolments in each cohort that I teach are:

- <50 (1)
- 51-100 (4)
- 101-150 (5)
- 151-200 (6)
- 201-250 (7)
- >250 (3)

My highest earned academic degree is:

- PhD (2)
- MBBS (4)
- Master (5)
- Baccalaureate (6)
- Other: please enter your degree (7) ________________________________________________

I have been teaching pharmacology for:

- Less than 3 years (1)
- 3-5 years (2)
- 6-10 years (3)
- 11-20 years (4)
- >20 years (5)

I identify as:

- Male (1)
- Female (2)
- Prefer not to respond (5)
- Other (4) ________________________________________________

Your views on Core Concepts           Imagine your current/recent pharmacology students three to five years after their graduation.  What few essential core concepts would you expect them to remember, understand deeply, and apply effectively in their professional work?   Please list a few – ideally between 3 and 7 – core concepts that are foundational for pharmacology students in the text box below. Feel free to write as much or as little as you wish about your core concepts. Your draft concepts should be Your draft concepts should be **big ideas** that are **useful** to solve problems and **enduring**, and they should **not** be **topics or facts.**

________________________________________________________________

**What is the main source of the core concepts currently taught in your courses? In other words, where do most of the big essential ideas that you teach come from? Choose all applicable**

- My own education (1)
- My professional experience (2)
- My research (3)
- Professional bodies / guidelines (4)
- Accrediting bodies (5)
- Textbook(s) (6)
- My school / college / university (7)
- Other (describe) (8) ________________________________________________

All of us on the PharmAlliance Core Concepts research team sincerely thank you for your valuable time and collaboration on this project. We have a list of concepts that other pharmacology educators have suggested to be core concepts in our small first round of survey. Would you be prepared to spend around 5 minutes to provide feedback on our draft list? If not, you will move to the end of the survey

- Yes (1)
- No thanks, please end the survey now (2)

Consider the following list. Please tick any terms that you think ARE suitable and should be included as core concepts.

- Adherence (52)
- Affinity (4)
- Bioavailability (6)
- Cellular physiology (7)
- Clearance (9)
- Concentration-response / potency (10)
- Dissolution (11)
- Distribution (12)
- Drug effects on homeostasis (13)
- Drug interactions (14)
- Drug target (48)
- Efficacy (15)
- Enzyme kinetics (50)
- Excretion (18)
- Half life (19)
- Homeostasis (20)
- Hydrophobicity (22)
- Immunogenicity (23)
- Central dogma: Macromolecules / DNA/RNA/protein (26)
- Mechanism of drug action (27)
- Metabolism (29)
- Organ systems / tissues / cells (30)
- Partition coefficient (31)
- Pathophysiology (32)
- Permeability (33)
- Pharmacodynamics (34)
- Pharmacogenetics (35)
- Pharmacokinetics (36)
- Renal function and clearance (38)
- Signalling (39)
- Solubility (40)
- Specificity and selectivity (41)
- Stability (42)
- Statistical significance (51)
- Tolerance (43)
- Toxicity / ADR (44)
- Transcription / translation (45)
- Volume of distribution (46)

Q25 Please tick any terms that you think are NOT suitable and should be removed.

- Adherence (52)
- Affinity (4)
- Bioavailability (6)
- Cellular physiology (7)
- Clearance (9)
- Clinical response (48)
- Concentration-response / potency (10)
- Dissolution (11)
- Distribution (12)
- Drug effects on homeostasis (13)
- Drug interactions (14)
- Drug target (47)
- Efficacy (15)
- Enzyme kinetics (49)
- Excretion (18)
- Half life (19)
- Homeostasis (20)
- Immunogenicity (23)
- Central dogma: Macromolecules / DNA/RNA/protein (26)
- Mechanism of drug action (27)
- Metabolism (28)
- Organ systems / tissues / cells (30)
- Pathophysiology (32)
- Permeability (33)
- Pharmacodynamics (34)
- Pharmacogenetics (35)
- Pharmacokinetics (36)
- Renal function and clearance (38)
- Signalling (39)
- Solubility (40)
- Specificity and selectivity (41)
- Stability (42)
- Statistical significance (51)
- Tolerance (43)
- Toxicity / ADR (44)
- Transcription / translation (45)
- Volume of distribution (46)

 Only aggregate summaries of this survey data will ever be reported and published.      Any and all individual data will be de-identified prior to analysis.*That said, if you would like to receive reports on this core concepts research project, please feel free to insert your email address below[TA1] .*

________________________________________________________________

Q26 We would like your input to help to improve the survey. Please answer the questions below in the text box. Thanks so much! 
How long did it take you?·        Were there parts you didn’t understand? If so, please explain·        What was missing?·        Did the categories / options work for you? If not, why not?

________________________________________________________________

End of Block: Second Response block
